# Analysis of the static and dynamic characteristics of the electro-hydraulic pressure servo valve of robot

**DOI:** 10.1038/s41598-023-42860-1

**Published:** 2023-09-20

**Authors:** Jianrui Zhang, Xiaonan Pan, Jinchang Guo, Jianxiao Bian, Jian Kang

**Affiliations:** 1 College of Intelligent Manufacturing, Longdong University, Qingyang, China; 2 College of Automation, Northwestern Polytechnical University, Xi’an, China

**Keywords:** Mechanical engineering, Design, synthesis and processing

## Abstract

In this study, we comprehensively investigate the structure and operational principles of the Rotary Direct Drive Electro-Hydraulic Pressure Servo Valve (RDDPV). Our objective is to establish the dynamics equations governing the motor, slide valve, and bias mechanism of the valve. Additionally, we construct a mathematical model for the servo valve controller, while ensuring the linearization of the controller model. Furthermore, we conduct an in-depth analysis of the static characteristics of the valve, including linearity, dead zone, hysteresis loop, and zero drift. Regarding the dynamic characteristics, we establish a dynamic mathematical model for the RDDPV valve. Subsequently, we subject the servo valve to analysis with a focus on frequency response and dynamic response, using the control current as the input and the pressure as the output. To perform these analyses, we employ the software package SIMULINK of MATLAB, facilitating dynamic simulations. Remarkably, the simulation results exhibit the valve's conformity to design requirements, underscoring its suitability for subsequent research and development endeavors. Through our rigorous investigation, we offer essential technical support for the forthcoming stages of the valve's research and development, thereby laying a robust foundation for its further advancement.

## Introduction

The rotary direct drive electro-hydraulic pressure control servo valve (hereinafter referred to as RDDPV)^1,2^ converts the rotary motion of torque motor into the linear motion of power spool via the eccentric drive mechanism, so as to change the throttling area ratio of the oil inlet and return windows, and output the corresponding load pressure; servo control is achieved via the closed-loop electrical feedback of motor position and output pressure. Klarecki analyzed the influence of electronic controller parameters on the dynamic characteristics of electro-hydraulic servo system^3^. With respect to related researches in China, Songjing et al.^4^ studied the vibration noise of torque motor, and in their work, the self-excited vibration of the torque motor is suppressed by adding magnetic fluid to the working clearance. At present, there are relatively few researches on the dynamic characteristics of rotary direct drive electro-hydraulic pressure servo valve.

In response to the imperative of further reducing size to facilitate integration within narrow servo control systems, such as aircraft engine control, ongoing efforts both domestically and internationally have sought to improve motor configurations, slide valve movement, and mechanical drive interfaces^5^. Notably, in 1966, IBM pioneered the voice coil motor^6^, which subsequently found application in the direct drive of hydraulic slide valves by companies like Parker. Additionally, piezoelectric ceramics, renowned for their high energy density and force output, have progressively been integrated into direct-drive valves. To address the limited output displacements of piezoelectric ceramics, Beihang proposed a compact hydraulic displacement amplification structure, significantly enhancing slide valve stroke in limited space, thereby augmenting control flow rate and response frequency of the direct-drive valve^7^.

Another innovation was introduced by a study^8^, proposing a rotary valve-type direct-drive valve that effectively reduces the hydrodynamic force during spool motion by controlling the throttle port size through rotary motion of the slide valve. In a separate development, Zhejiang University of Technology introduced a fast response 2D valve, driven by a stepper motor to rotate the slide valve. The high and low-pressure holes, along with spiral grooves on the shoulder of the spool, form a hydraulic resistance half-bridge, enabling precise servo control of the slide valve's horizontal position^9^. Currently employed in missile servo control, it is noteworthy that the 2D valve’s spool drive follows the same configuration as traditional two-stage valves with the same hydraulic pressure. Consequently, its response frequency and zero characteristics are susceptible to drift due to changes in external factors such as oil supply pressure and temperature.

In the 1990s, a novel structure called the Rotary Direct Drive Electro-Hydraulic Pressure Control Servo Valve (RDDPV) emerged, featuring diverse rotary drive mechanisms that convert motor torque into valve torque and further to servo valve torque^10^. Notably, these rotary drive mechanisms facilitate the conversion of motor rotarymotion into linear motion of the slide valve, with the motor rotation and slide valve translation direction arranged perpendicular to the direction of the servo valve. This structural arrangement renders the linear direct-drive valve more compact and less sensitive to external vibrations affecting the slide valve’s movement. Woward company has successfully commercialized rotary direct-drive valves based on this principle. Internationally, rotary direct-drive valves have found progressive applications in aircraft electronic anti-skid brakes, aircraft rudder and engine control, and other fields.

Despite these advancements, the development of rotary direct-drive electro-hydraulic servo valves remains in a nascent stage. The selection of structural parameters, particularly those at the drive interface, still lacks clarity, and a theoretical basis for design and manufacture is yet to be fully established.

## Structure and working principle of RDDPV valve

**Figure 1:**
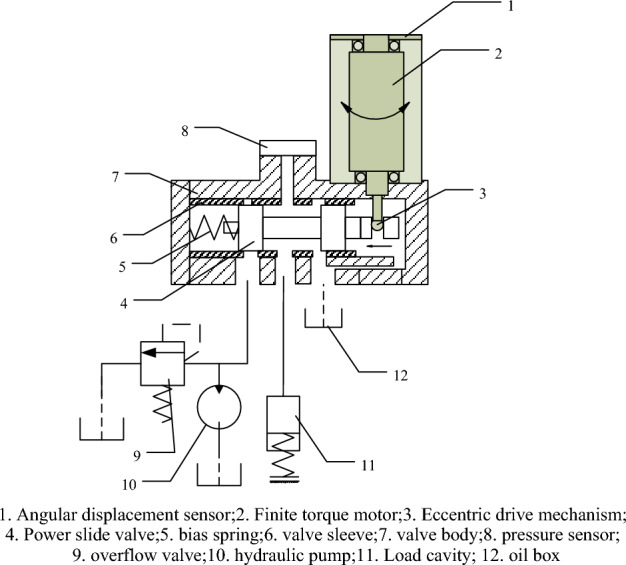
Schematic diagram of the working principles of RRDDPV.

The working principle of rotary direct drive electro-hydraulic pressure servo valve (RDDPV) is illustrated in Fig. 1, and it mainly consists of electronic controller, limited angle torque motor, eccentric drive mechanism, slide valve pair and related sensors. When the input command of the electronic controller is 0, the limited angle torque motor has no torque output, at this time, the slide valve is pushed to the far right end by the reset spring, the oil inlet is closed, the working chamber and the oil return port are connected, and the output pressure of servo valve is zero; when a non-zero positive command signal ${{\varvec{i}}}_{0}$ is input, the electronic controller performs calculation and outputs a PWM signal to drive the limited angle torque motor to rotate, and the eccentric drive mechanism converts the rotational motion of motor into the linear motion of power spool; the power slide valve is in the form of under lap, and the linear movement of spool changes the throttling area ratios of the oil inlet and return ports; the load cavity connected to the ${{\varvec{P}}}_{{\varvec{c}}}$ port is a closed sealed cavity during braking, so the steady-state pressure of load cavity only changes with the throttling area ratios of the oil inlet and return ports; the effective stroke of spool is the amount of under lap^11,12^. The valve adopts the form of electric feedback for servo control; the angle displacement sensor feeds back the rotation angle of torque motor to the sensor to form a closed-loop of motor position; the pressure sensor feeds back the pressure of the working chamber to provide external closed-loop control of pressure^13^.

**Figure 2:**
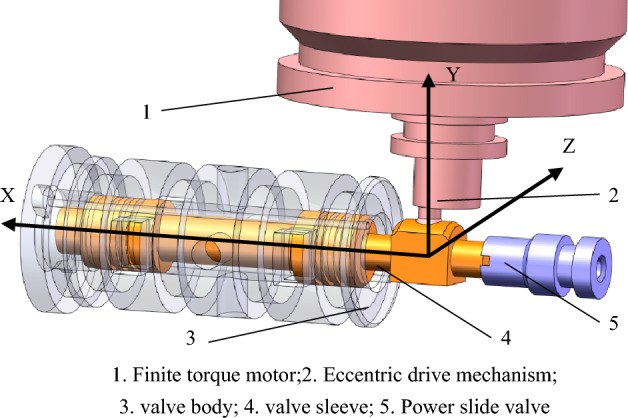
3D structural diagram of eccentric mechanism at the end of motor shaft.

Figure 2 shows the schematic diagram of the initial state of the eccentric drive mechanism at the end of motor shaft. In Fig. 2, the ball and the cylindrical hole have the same nominal size, the origin O of the coordinate system is the intersection between the central axis of slide valve and the central axis of cylindrical hole, the X axis passes through O and is parallel to the central axis of slide valve, and the Y axis passes through O and is parallel to the motor shaft. The motor shaft is ensured to be on the YZ plane via structural and size adjustment, and the distance between the ball center and the XOZ plane is *h*.

In the initial state, the central axis of the cylindrical hole coincides with the Y axis; when the motor rotates, the ball center rotates around the motor axis, and its movement can be decomposed into movement in the X and Z directions; the ball's movement in the X direction drives the spool to translate, thereby changing the throttling area of the oil inlet and return ports; the movement in the Z direction drives the spool to rotate around the X axis.

## Mathematical model of the RDDPV valve

### Kinetic equation of motor

The motor rotor is under the electromagnetic driving torque of^14^:

1 $${T}_{em}={k}_{t}{i}_{0}-{k}_{m}{\alpha }^{2}$$

where ${i}_{0}(\mathrm{A})$ is the input current of the motor; $\alpha (\mathrm{rad})$ is the rotation angle of the motor rotor; ${k}_{t}$ is the current torque coefficient; ${k}_{m}$ is the angle torque coefficient.

The kinetic equation of the motor rotor is^15^:

2 $${T}_{em}={J}_{r}\ddot{\alpha }+{B}_{r}\dot{\alpha }+{T}_{f}$$

where ${T}_{f} (\mathrm{N}\cdot \mathrm{m})$ is the load torque; $J_{r} ({\text{kg}} \cdot {\text{m}}^{2} )$ is the moment of inertia of the motor rotor; ${B}_{r}$ is the damping coefficient of the motor rotor.

### Kinetic equation of slide valve

During the opening process, the slide valve has movement in two directions: translation along the axial direction of slide valve and rotation around the central axis of slide valve. By decomposing the movement in two directions, we have:

3 $${F}_{x}={m}_{v}{\ddot{x}}_{v}+{B}_{v}{\dot{x}}_{v}+{k}_{v}\left({x}_{v}+{x}_{v0}\right)+{F}_{s}+{F}_{f}$$

4 $${F}_{s}=2{C}_{d}\pi {D}_{v}\mathrm{cos}\varphi \left[{x}_{v}\left({P}_{s}-{P}_{c}\right)-\left(U-{x}_{v}\right)\left({P}_{c}-{P}_{0}\right)\right]$$

5 $${T}_{\beta v}={J}_{\beta v}{\ddot{\beta }}_{v}+{B}_{\beta v}{\dot{\beta }}_{v}$$

where ${F}_{x}(\mathrm{N})$ is the driving force for the axial translation of the slide valve; ${m}_{v}(\mathrm{kg})$ is the spool mass; ${x}_{v}(\mathrm{m})$ is the displacement of the slide valve; ${B}_{v}$ is the axial translation damping coefficient of the slide valve; ${k}_{v}(\mathrm{kN}/\mathrm{m})$ is the stiffness of bias spring; ${x}_{v0}(\mathrm{m})$ is the pre-compression amount of the bias spring; ${F}_{s}(\mathrm{N})$ is the hydraulic force; ${F}_{f}(\mathrm{N})$ is the friction force; ${C}_{d}$ is the flow coefficient at the port of slide valve; ${D}_{v}(\mathrm{m})$ is the diameter of the spool end; $\varphi (\mathrm{rad})$ is the jet angle of the slide valve orifice; $U(\mathrm{m})$ is the pre-opening amount of the slide valve; ${P}_{s}(\mathrm{MPa})$ is the oil supply pressure; ${P}_{c}(\mathrm{MPa})$ is the brake pressure; ${P}_{0}(\mathrm{MPa})$ is the oil return port pressure;${T}_{\beta v}(\mathrm{N}\cdot \mathrm{m})$ is the driving torque for the rotation of slide valve; ${J}_{\beta v}(\mathrm{kg}\cdot {\mathrm{m}}^{2})$ is the moment of inertia of the slide valve to its central axis; ${\beta }_{v}(\mathrm{rad})$ is the rotation angle of the spool around the spool axis; ${B}_{\beta v}$ is the rotational damping coefficient of the slide valve.

In the slide valve, the spool has clearance fit with the valve sleeve. Due to the pressure difference between the two ends of spool, the liquid in the clearance will form asymmetric pressure distribution from the high pressure end to the low pressure end, which can be called the pressure on the slide valve side. According to the slit flow theory of fluid mechanics, the pressure on the slide valve side changes with the clearance size. If the clearance changes symmetrically along the longitudinal axis of the spool, the resultant pressure on the slide valve side is zero. However, when the clearance changes asymmetrically along the longitudinal axis of the spool, the resultant pressure on the side slide valve side is not zero, and the side pressure friction is formed with the movement of the spool.

Therefore, asymmetrical pressure on the slide valve side can be caused by non-concentric spool and sleeve or asymmetrical clearance due to various reasons such as machining accuracy or assembly, which will further lead to side pressure friction. If the pressure on slide valve side further acts on the spool surface and makes it eccentric, the clearance will become even more asymmetrical, and the pressure on the spool side will increase. In severe cases, the oil film between the spool and valve sleeve will be destroyed, resulting in dry friction, which significantly increases the friction between the spool and sleeve, resulting in “hydraulic clamping” and equipment failure. The friction force can be represented as^6^:

6 $${F}_{f}=\frac{\pi }{4}LD\Delta P$$

where $L(\mathrm{m})$ is the spool shoulder width, and $D(\mathrm{m})$ is the spool diameter, $\Delta p(\mathrm{MPa})$ is the pressure difference between the two ends of the spool.

The steady-state hydraulic force is the hydraulic force on the spool due to changes of flow rate and direction when fluid flows into the valve cavity and passes through the control window of the valve, and it is always in the direction trying to close the control window of the slide valve. According to the momentum theorem, the steady-state hydraulic force is:

7 $${F}_{s}=0.43W{x}_{v}({P}_{h}-{P}_{s})$$

where $W(\mathrm{m})$ is the width of slide valve window, ${x}_{v}(\mathrm{m})$ is the spool displacement, ${P}_{h}(\mathrm{MPa})$ is the pressure at the inlet of slide valve cavity, and ${P}_{s}(\mathrm{MPa})$ is the brake pressure.

The transient hydraulic force is generated by the acceleration of fluid. Assuming that the oil cannot be compressed, and the oil quality in the valve cavity remains unchanged, the change rate of oil speed in the valve cavity is $\mathrm{d}v/\mathrm{d}t$, the oil acceleration force is^16^:

8 $$F=m\frac{dv}{dt}$$

Set the axial distance between the inlet and outlet of the slide valve as $L(\mathrm{m})$, the oil density as $\rho ({\mathrm{kg}/\mathrm{m}}^{3})$, and $Q(\mathrm{L}/\mathrm{s})$ is the flow rate in the valve cavity. Then, Eq. (7) can be modified to:

9 $$F=\rho L\frac{dQ}{dt}$$

According to the continuity equation, the flow in the valve cavity is the flow at the valve port, and the throttling formula can be obtained:

10 $$Q={C}_{v}W{x}_{v}\sqrt{\frac{2\Delta P}{\rho }}$$

where ${C}_{v}$ is the flow coefficient; $\Delta p(\mathrm{MPa})$ is the pressure difference between the inlet and outlet of the valve cavity.

According to the above Equation, we can obtain:

11 $$F={C}_{v}WL\sqrt{2\rho \Delta P}\frac{\mathrm{d}{x}_{v}}{\mathrm{d}t}+\frac{L{C}_{v}W{x}_{v}}{2\sqrt{\Delta P/\rho }}\frac{\mathrm{d}(\Delta P)}{\mathrm{d}t}$$

In general, the impact of $\mathrm{d}(\Delta p)/\mathrm{d}t$ on the transient hydraulic force is very small, which can be ignored. The transient hydraulic force is constantly in the opposite direction of $F$, and the transient hydraulic force is:

12 $${F}_{t}=-{C}_{v}WL\sqrt{2\rho \Delta P}\frac{\mathrm{d}{x}_{v}}{\mathrm{d}t}$$

### Kinematic and mechanical equations of eccentric mechanism

**Figure 3:**
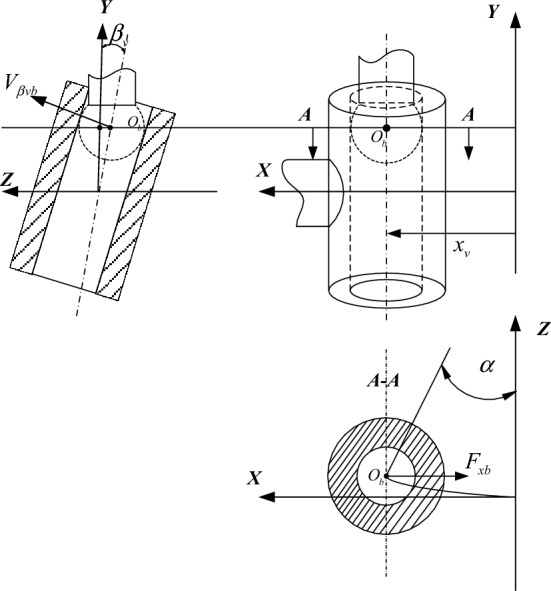
Schematic diagram of eccentric mechanism operation.

The motor rotates and drives the eccentric mechanism, the eccentric mechanism pushes the spool to move linearly, and the eccentric ball has eccentric rotation, which also drives the spool to rotate around its axis. Therefore, the spool movement can be decomposed into linear movement along the X axis and rotation around the X axis. As shown in Fig. 3, the displacement of linear movement is *x*_*v*_ $(\mathrm{m})$, and the rotation angle of the spool around the spool axis is ${\beta }_{v}(\mathrm{rad})$. The kinematics between the spool movement and the motor angle $\alpha$ can be represented as^17^:

13 $${x}_{v}=e\,\mathrm{sin}\,\alpha $$

14 $$\mathrm{tan}{\beta }_{v}=\frac{e\left(1-\mathrm{cos}\,\alpha \right)}{h}$$

where *h* is the distance from the center of the ball to the plane, *e* is the eccentricity distance between the eccentric shaft and the motor rotation center,

According to Fig. 3, the ball are under the spool forces of ${F}_{xb}(\mathrm{N})$ and ${F}_{\beta vb}(\mathrm{N})$, and we have

15 $${F}_{xb}={F}_{x}$$

16 $${T}_{\beta v}={F}_{\beta vb}\frac{h}{\mathrm{cos}\,{\beta }_{v}}$$

17 $${T}_{f}={F}_{xb}e\,\mathrm{cos}\,\alpha +{F}_{\beta vb}e\,\mathrm{sin}\,\alpha\, \mathrm{cos}\,{\beta }_{v}$$

### Mathematical model of controller

The main functions realized by controller include signal conditioning, closed-loop pressure control, feedback control of motor angle and PWM amplification. Among them, signal conditioning is to convert the input control current signal ${i}_{i}$ into the voltage signal ${u}_{i}$, and perform noise reduction filtering processing. The closed-loop pressure control adopts PID control to convert the hydraulic pressure ${P}_{c}$ into the corresponding voltage, and the voltage signal is linearly amplified by the amplifier and used as the pressure feedback signal ${u}_{p}$. According to the difference between the input signal and the pressure feedback signal, the proportional, integral and differential operations are performed to obtain the output signal ${u}_{m}$ of pressure control. An angular displacement sensor is installed in the angle feedback control of motor, which converts the motor angle $\alpha$ into the corresponding voltage. The voltage signal is linearly amplified by the amplifier and used as the angle feedback signal ${u}_{\alpha }$ of motor. The proportional coefficient of this process is the motor angle electric feedback coefficient. The difference between the output signal ${u}_{m}$ of pressure feedback control and the feedback signal ${u}_{\alpha }$ of motor angle is obtained, and the final motor control signal is output. The control signal of motor requires power amplification, and PWM power amplification is used to amplify the signal by ${K}_{PWM}$. In summary, the mathematical model of the controller part is^18^:

18 $${u}_{i}=k{i}_{i}$$

19 $${u}_{m}=\left({u}_{i}-{P}_{c}{k}_{f2}\right)\left({K}_{p}+{K}_{I}\frac{1}{s}+{K}_{D}s\right)$$

20 $${u}_{0}=\left({u}_{m}-\alpha {k}_{f1}\right){K}_{PWM}$$

### Local linearization of the mathematical model of RDDPV

In the actual working condition of RDDPV, the displacement of the spool is very small, which is around 0.1 × 10^−3^ m. According to Eq. (12), the rotation angle of the motor is 4.78°, so Eqs. (12) and (13) can be linearized to

21 $${x}_{v}=e\alpha $$

22 $${\beta }_{v}=\frac{e{\alpha }^{2}}{2h}={k}_{\beta v}\alpha $$

To sum up, the kinetic equation of the motor rotor at the operating point can be obtained as:

23 $${T}_{em}=\left({J}_{r}+{m}_{v}{e}^{2}+{J}_{\beta v}eh\alpha {k}_{\beta v}\right)\ddot{\alpha }+\left({B}_{r}+{B}_{v}{e}^{2}+{B}_{\beta v}eh\alpha {k}_{\beta v}\right)\dot{\alpha }+\left({k}_{v}+{k}_{s}\right){e}^{2}\alpha +{k}_{v}e{x}_{v0}$$

## Analysis of the static characteristics of the RDDPV valve

Considering the oil compressibility, the output brake pressure of slide valve and the spool displacement has the relationship of^19^:

24 $$ \left\{ {\begin{array}{*{20}c}    {q_{s}  = C_{v} Wx_{v} \sqrt {\frac{2}{\rho }\left( {P_{h}  - P_{s} } \right)} } & {x_{v}  > 0}  \\    {q_{s}  = C_{v} Wx_{v} \sqrt {\frac{2}{\rho }\left( {P_{s}  - P_{r} } \right)} } & {x_{v}  \le 0}  \\   \end{array} } \right. $$

25 $${q}_{s}=\frac{{V}_{t}}{E}s{P}_{s}$$

where *E* = 1000 MPa is the bulk modulus of oil, and ${V}_{t}=0.4 \mathrm{L}$ is the volume of the brake cavity.

**Figure 4:**
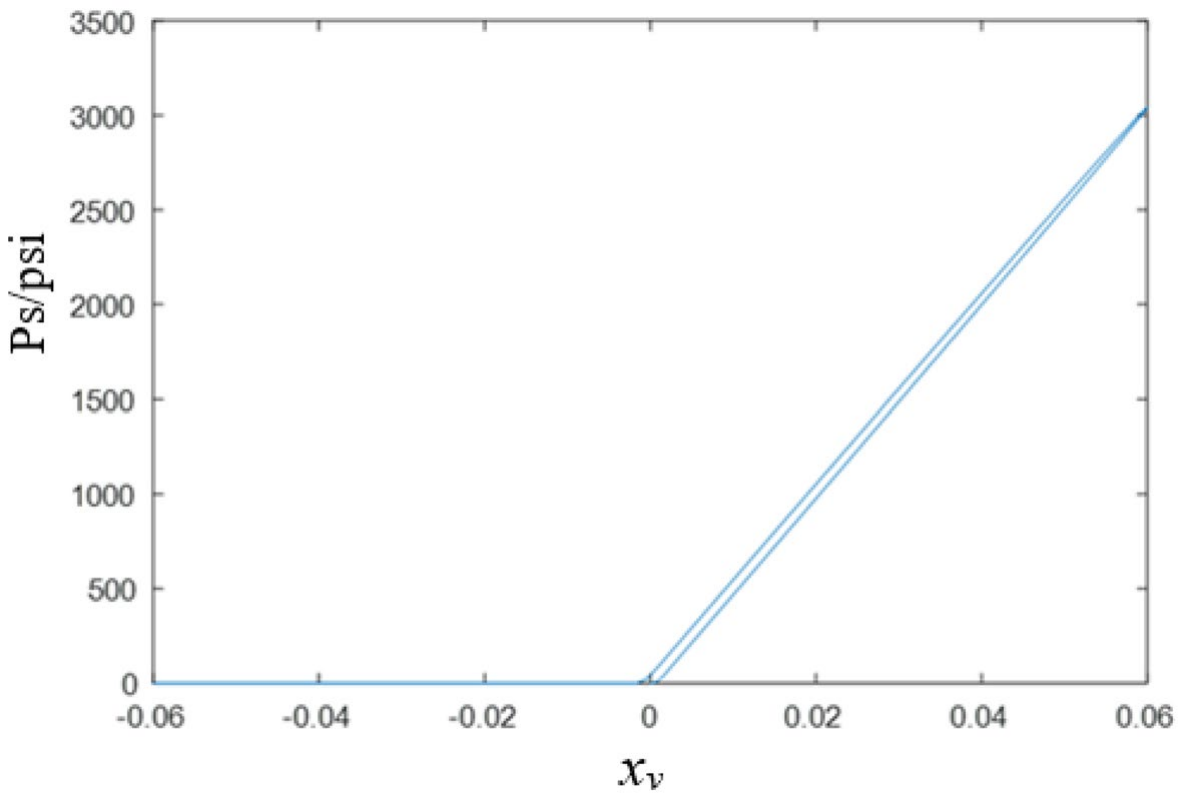
Static characteristic curve of RDDPV.

The static characteristic curve of RDDPV is as shown in Fig. 4.

### Linearity

The ratio of the maximum deviation of the nominal pressure curve from the nominal pressure gain profile to the rated current is called linearity.

**Figure 5:**
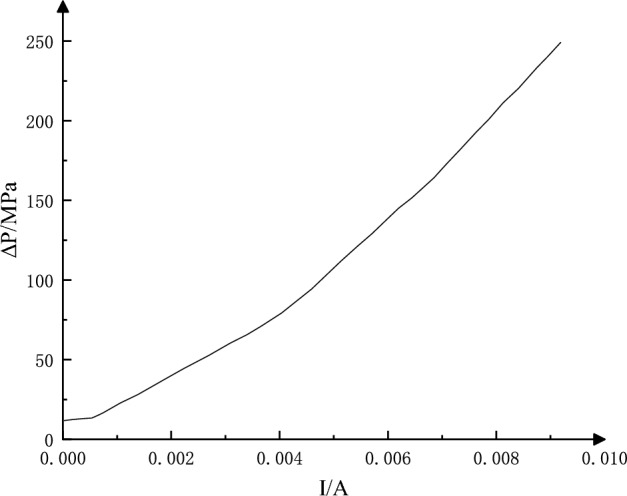
Linearity analysis curve of DDPV.

According to the linearity analysis curve in Fig. 5, the linearity of RDDPV is 0.67%.

### Dead zone

When the input signal (current signal) of the electro-hydraulic servo valve changes, the spool must overcome certain resistance to have displacement^20^. Therefore, when the differential current only changes slightly, the spool will not have displacement (or the output flow of the electro-hydraulic servo valve does not change). This small variation range of differential current not sufficient to change the output signal is called the static characteristic dead zone of the servo valve. According to the simulation results of static characteristics, the dead zone of the RDDPV valve can be obtained, which about 0.4 × 10^−3^A.

### Hysteresis

**Figure 6:**
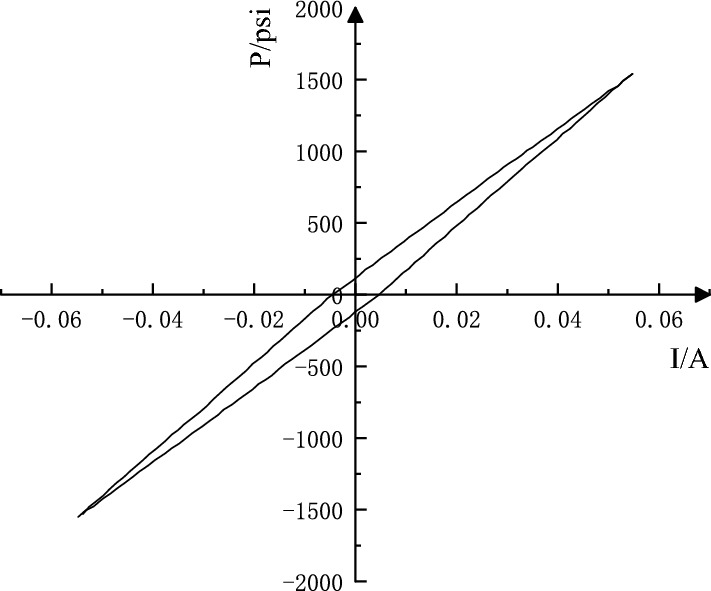
Hysteresis analysis curve of RDDPV.

Between positive and negative rated currents, the percentage of the maximum difference between the two currents that produce the output pressure of the system and the rated current is called hysteresis when cycling at a speed that does not dynamically work^21^. The hysteresis analysis curve is shown in Fig. 6.

26 $$Hysteresis=\frac{0.01078-0.009145}{0.06}\times 100\%=2.73\%$$

The hysteresis of the servo valve is caused by the magnetic hysteresis of the force (torque) motor and the oil clearance of the valve. The oil clearance of the valve is caused by friction and the clearance of the mechanical fastening part. The hysteresis loop value changes with the current, when the current is small, the hysteresis decreases with constant amplitudes, so hysteresis generally won't not affect the system stability. The loop value caused by the oil clearance is a fixed value, and the oil clearance will significantly increase when the oil dirty, which might cause system instability.

### Zero drift

**Figure 7:**
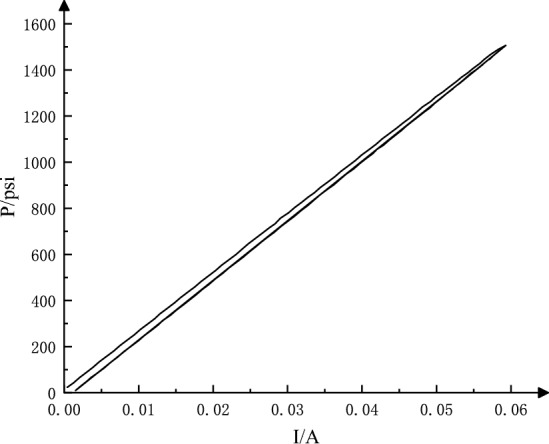
Zero drift analysis curve of RDDPV.

The servo valve is adjusted under the specified test conditions according to the test standards. When the working conditions (oil supply pressure, oil return pressure, working oil temperature, zero current, etc.) change, the zero position of the valve will shift. The ratio of the change of zero-bias current caused by changes in working conditions such as pressure and temperature to the rated current is called zero drift. When the oil supply pressure is changed to 11 MPa, the static characteristic curve is as shown in Fig. 7.

27 $$\mathrm{Zero drift}=\frac{0.0006228-0.0006002}{0.06}\times 100\%=0.038\%$$

## Analysis of the dynamic characteristics of the RDDPV valve

**Figure 8:**
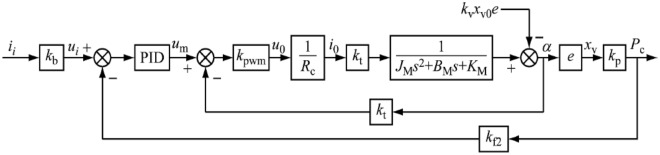
Transfer function block diagram of the RDDPV pressure.

Combining the equation, the transfer function block diagram of the RDDPV pressure servo valve can be obtained, as shown in Fig. 8.

PID control of the pressure external closed loop. Pressure sensor (response frequency > 1 kHz, considered as a proportional link) will be converted into the corresponding voltage by the hydraulic pressure ${P}_{c}$, the voltage signal by the amplifier linear amplification as the pressure feedback signal ${u}_{P}$, the proportionality coefficient of this process for the ${k}_{f2}$ (i.e., ${u}_{P}={P}_{c}{k}_{f2}$), known as the load pressure electrical feedback coefficient; according to the input signal ${u}_{i}$ and the difference between the pressure feedback signal ${u}_{P}$ for the proportionality, integration and differentiation operations, to obtain the pressure feedback control output signal ${u}_{m}$. This PID control methodology enables precise and efficient pressure regulation in the external closed-loop system. It holds great promise for a wide range of applications where accurate pressure management is crucial.

The main structural parameters of RDDPV are listed in Table 1.

**Table 1:** Main structural parameters of RDDPV.

Parameter	Value	Parameter	Value
*t*_*e*_ (s)	1.6 × 10^−3^	*k*_*t*_ (N·m·A^−1^)	0.05
*D*_*v*_ (m)	6 × 10^−3^	*U* (m)	0.1
*m*_*v*_ (kg)	5 × 10^−3^	*J*_*βv*_ (g·m^2^)	1.6 × 10^−3^
*x*_*v0*_ (m)	1 × 10^−3^	*k*_*v*_ (kN·m^−1^)	6
*h* (m)	1.6 × 10^−3^	*e* (m)	1.210^−3^
*R*_*c*_ (Ω)	23.3	*V* (L)	10 × 10^−3^

In Table 1, *t*_*e*_ is motor mechanical constant,*D*_*v*_ is the diameter of the spool end face, *m*_*v*_ is the spool mass, *x*_*v0*_ is the pre-compression of the reset spring, *h* is the distance from the center of the ball to the plane, *R*_*c*_ is the motor internal resistance, *k*_*t*_ is the current moment coefficient, *U* is the slide valve pre-opening, *J*_*β*_v is the spool rotational inertia, *k*_v_ is the reset spring stiffness, *e* is the eccentricity distance between the eccentric shaft and the motor rotation center, *V* is the load chamber volum.

The dynamic characteristics of a valve are generally represented using the frequency response. The frequency response of the RDDPV valve is the complex ratio of the output displacement of the slide valve to the current when the input current has constant-amplitude variable-frequency sinusoidal variation within a certain frequency range^22^. A dynamic mathematical model is constructed based on the analysis of the RDDPV valve. The frequency response and dynamic response of the servo valve are analyzed with the control current as the input and the pressure as the output. The software package SIMULINK of MATLAB is used for dynamic simulation.

**Figure 9:**
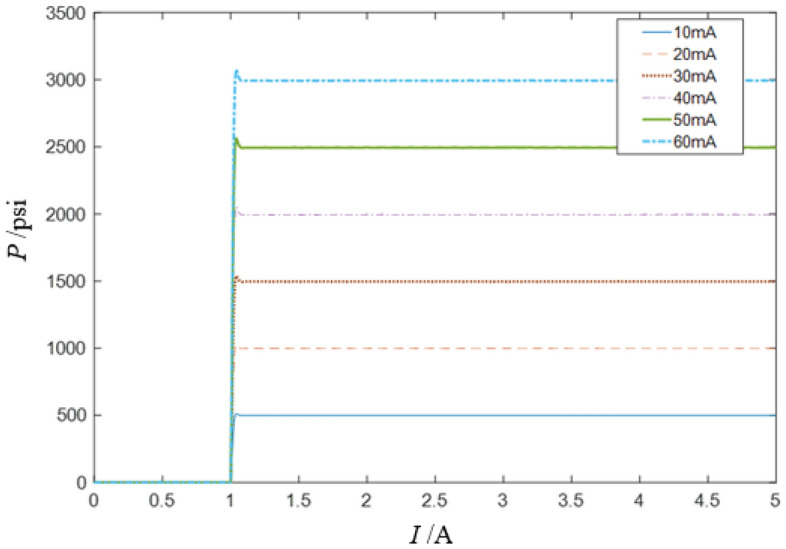
Step response curve of RDDPV pressure servo valve.

The step response is used to illustrate the transient response of the valve. The step response is the tracking process of the step input current by the output pressure when the load pressure is zero under the rated pressure. Considering the step response of the RDDPV valve, when the oil supply pressure is 3000psi, the control current of 10 mA to 60 mA is input, and the step response curve is obtained, as shown in Fig. 9.

**Figure 10:**
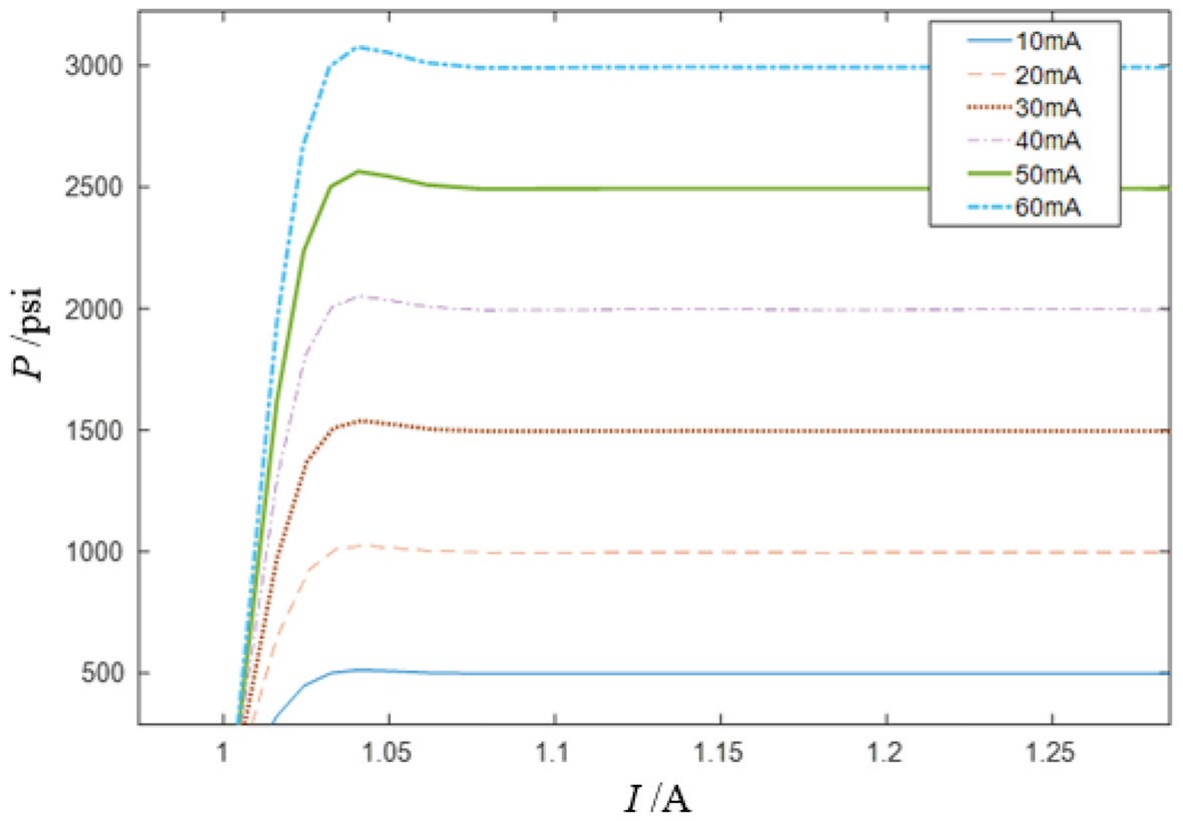
Partial enlarged diagram of the step response of RDDPV pressure servo valve.

When the input current amplitude is in the range of 0.01A to 0.06A, the step response is mainly analyzed. As shown in Fig. 10, when current is input to the servo valve at 1 s, the displacement response of the brake pressure reaches a stable value within 1.08 s. The rise time, peak time and overshoot of the system in the initial stage are shown in the diagram, in which, the rise time is 0.08 s, the overshoot is 3.3%, and the peak time is 0.0817 s.

## Conclusions

This study delved into the structure and operational principles of the Robot Rotary Direct Drive Electro-Hydraulic Pressure Servo Valve (RDDPV). By establishing dynamic models for the motor, slide valve, and eccentric mechanism within the valve, as well as a mathematical model for the controller, we comprehensively examined various static characteristics of the valve, including linearity, dead zone, hysteresis, and zero drift.

Furthermore, leveraging the dynamic mathematical model of the RDDPV valve, we conducted in-depth analyses of the frequency response and dynamic response of the servo valve, employing the control current as the input and pressure as the output. To achieve this, we utilized Simulink in the Matlab software package for dynamic simulation. The simulation results demonstrated that all performance indicators satisfactorily met the specified requirements, thus laying a robust foundation for subsequent analyses pertaining to reliability, durability, and anti-contamination.

Through our rigorous investigation, we have provided a comprehensive understanding of the RDDPV system's behavior and validated its efficacy in meeting operational criteria. The insights gained from this study contribute to the optimization and further advancement of rotary direct drive electro-hydraulic pressure servo valves for various industrial applications.

## Data Availability

All data generated or analysed during this study are included in this published article.
